# Cholesterol levels and development of cardiovascular disease in Koreans with type 2 diabetes mellitus and without pre-existing cardiovascular disease

**DOI:** 10.1186/s12933-019-0943-9

**Published:** 2019-10-22

**Authors:** Mee Kyoung Kim, Kyungdo Han, Han Na Joung, Ki-Hyun Baek, Ki-Ho Song, Hyuk-Sang Kwon

**Affiliations:** 1 Division of Endocrinology and Metabolism, Department of Internal Medicine, Yeouido St. Mary’s Hospital, College of Medicine, The Catholic University of Korea, 10 63-ro, Yeongdeungpo-gu, Seoul, 07345 Republic of Korea; 2 Department of Medical Statistics, College of Medicine, The Catholic University of Korea, Seoul, 06591 Republic of Korea

**Keywords:** Cholesterol, Cardiovascular disease, Diabetes mellitus, Dyslipidemia, Korea

## Abstract

**Background:**

The aim of the present study was to identify a threshold for the cholesterol level at which the risk of cardiovascular disease (CVD) begins to increase in people with type 2 diabetes mellitus (DM).

**Methods:**

Using the Korean National Health Insurance Service database, 2,077,135 people aged ≥ 40 years with type 2 DM who underwent regular health checks between 2009 and 2012 were included. Subjects with previous CVD were excluded. Cox regression analyses were performed to estimate the risk of CVD for each low-density lipoprotein cholesterol (LDL-C) group using the < 70 mg/dL as the reference group.

**Results:**

There were 78,560 cases of stroke (3.91%), and 50,791 myocardial infarction (MI, 2.53%) during a median follow-up of 7.1 years. Among participants not taking statins, LDL-C levels of 130–159 mg/dL and ≥ 160 mg/dL were significantly associated with the risk of MI: the hazard ratios (HRs) (95% confidence interval) were 1.19 (1.14–1.25) and 1.53 (1.46–1.62), respectively. Among participants taking statins, all categories of LDL-C level ≥ 70 mg/dL were significantly associated with increased risk of stroke and MI.

**Conclusions:**

We identified an increased risk of CVD in people with an LDL-C level ≥ 130 mg/dL among individuals with type 2 DM not taking statins. The risk of CVD was significantly higher in those taking statins with an LDL-C level ≥ 70 mg/dL.

## Background

Current evidence indicates that dyslipidemia is a strong predictor of cardiovascular disease (CVD). Lowering low-density lipoprotein cholesterol (LDL-C) level with statin therapy reduces incidence of major CVD [1–5]. In the recent guidelines, the cholesterol targets are based on several primary and secondary prevention statin trials that have shown improved outcomes with more intensive LDL-C lowering [1–3]. However, none of these trials used specific LDL-C targets to trigger adjustments in the medication dose, and LDL-C targets are usually extrapolated from these trials. The usefulness of the treat-to-target approach is controversial because of insufficient evidence. Recently, the standard versus intEnsive statin therapy for hypercholesteroleMic Patients with diAbetic retinopaTHY (EMPATHY) study demonstrated that, in patients with diabetic retinopathy, intensive statin therapy to achieve an LDL-C level < 70 mg/dL did not reduce incidence of the cardiovascular events compared with standard therapy to achieve levels of ≥ 100 and < 120 mg/dL [6].

Primary prevention research focuses on the thresholds for initiating treatment of modifiable risk factors and the optimal goals of risk factor modification. There is insufficient information available about the optimal cholesterol targets for preventing CVD development in people with type 2 diabetes mellitus (DM) [1–4]. We analyzed data from the Korean National Health Insurance System database (NHIS DB) and investigated the relationship between high cholesterol level and the onset of CVD in people with type 2 DM. Because the NHIS DB represents the entire Korean population, it has been used in a number of population-based nationwide studies of type 2 DM in Korea [7–12]. We classified the population into statin users and non-users, and we tried to identify a threshold for the cholesterol level at which the risk of CVD begins to increase in people with type 2 DM in each group.

## Methods

### Data sources

The National Health Insurance Service (NHIS) in the Republic of Korea covers about 97% of the Korean population and provides biennial health screening examinations, called the National Health Screening Program (NHSP) to all enrollees aged 40 years and older [7–12]. The NHIS includes an eligibility database (age, sex, socioeconomic variables, type of eligibility, etc.); a medical treatment database (based on the accounts submitted by medical service providers for medical expenses); a health examination database (results of general health examinations and questionnaires on lifestyle and behavior); a medical care institution database (types of medical care institutions, location, equipment, and number of physicians); and death information. The dates of death were obtained from the eligibility database, which was prepared by Statistics Korea [7–12].

### Study population

From January 1, 2009 to December 31, 2012, a total of 2,522,388 people aged ≥ 40 years with type 2 DM underwent a health examination. Among these, we selected participants who had been diagnosed as having type 2 DM. Patients were classified as having type 2 DM [7, 10]; (1) when they had at least one service claim with a diagnosis of type 2 DM based on ICD-10 (E11–14) in the outpatient or inpatient setting and were prescribed at least one antidiabetic drug at any time over 1 year to exclude prediabetic or non-diabetic individuals or (2) when they showed fasting plasma glucose 126 mg/dL during health examinations (newly diagnosed diabetes). For example, among those who had undergone a health examination in 2009, we selected participants who had at least one service claim with a diagnosis of type 2 DM based on ICD-10 (E11–14) and who were prescribed at least one antidiabetic drug at any time in 2009. The same criteria were used for 2010, 2011 and 2012, and we excluded duplicate individuals who underwent multiple health examinations in consecutive years. The index year was 2009–2012. We excluded 72,891 people with missing data for at least one variable. Ultimately, the study population was 2,209,704 people (Additional file 1: Figure S1). Because of the established association of cancer with low serum total cholesterol level, we excluded those with a history of cancer (n = 80,679). To avoid confounding by preexisting diseases and to minimize the possible effects of reverse causality, we also excluded those with a history of myocardial infarction (MI) or stroke (n = 291,683), as indicated by their medical treatment and health examination data before the index year. This study was approved by the Institutional Review Board of The Catholic University of Korea (No. SC18ZES10009). Anonymized and de-identified information was used for analyses and, therefore, informed consent was not obtained.

Covariates were based on the data from the index year and included age, sex, socioeconomic status (income level), body mass index (BMI; kg/m^2^), current smoking status, alcohol consumption, physical activity (no, yes), and systolic/diastolic blood pressure (mmHg). Blood samples for the measurement of serum glucose, creatinine, and lipid levels were drawn after an overnight fast. Blood samples for the measurement of total cholesterol, high-density cholesterol (HDL-C), and triglyceride (TG) levels were obtained at the health examination after the participant had fasted for at least 8 h. LDL-C levels were calculated from the Friedewald formula: LDL-C = total cholesterol − HDL-C − (TG/5). We excluded those with a TG level > 400 mg/dL (n = 71,935) in the analysis of LDL-C. In the analysis of non-HDL-C levels, we included all participants regardless of their TG level.

Regular exercise was defined as performing more than 30 min of moderate physical activity at least five times per week or more than 20 min of strenuous physical activity at least three times per week. Income level was dichotomized at the lowest 25%. We defined a statin user as a person who had been prescribed statins during 2009–2012.

### Study outcomes and follow-up

The end points of the study were newly diagnosed MI or stroke. MI was defined as hospitalization with ICD-10 MI diagnostic codes of I21 or I22. Stroke was defined as the recording of ICD-10 codes I63 or I64 during hospitalization with claims for brain magnetic resonance imaging or brain computed tomography. The study population was followed from baseline to the date of a cardiovascular event, or the time of the participant’s disqualification from receiving health services due to death or emigration, or until the end of the study period (December 31, 2017).

### Statistical analysis

Baseline characteristics are presented as the mean ± SD or n (%). Participants were divided into the following categories according to LDL-C level: < 70 mg/dL, 70–99 mg/dL, 100–129 mg/dL, 130–159 mg/dL, and ≥ 160 mg/dL. Participants were also divided into the following categories according to non-HDL-C level: < 100 mg/dL, 100–129 mg/dL, 130–159 mg/dL, 160–189 mg/dL, and ≥ 190 mg/dL. The incidence rate of primary outcomes was calculated by dividing the number of incident cases by the total follow-up duration (person-years). Cox regression analyses were performed to estimate the risk of CVD for each LDL-C group using the < 70 mg/dL group as the reference group and for each non-HDL-C group using < 100 mg/dL as the reference group. The American Association of Clinical Endocrinologists identified an additional “extreme high-risk” category for which very aggressive LDL-C lowering is recommended (< 55 mg/dL) [3]. We therefore performed an addition Cox regression analyses using the < 55 mg/dL group as the reference group. Participants were then classified into the following categories according to LDL-C level: < 55 mg/dL, 55–69 mg/dL, 70–99 mg/dL, 100–129 mg/dL, 130–159 mg/dL, and ≥ 160 mg/dL. To consolidate our findings, we analyzed the associations between lipid levels and CVDs using quintiles of LDL-C and non-HDL-C. A multivariable-adjusted proportional hazards model was applied that was adjusted for age, sex, BMI, smoking, alcohol consumption, regular exercise, household income, use of statins, fasting glucose levels, hypertension, and duration of diabetes. Sensitivity analyses excluding subjects with end points occurring within 2 year of follow-up were also performed. The potential effect modification by age and sex was evaluated through the stratified analysis and interaction testing using a likelihood ratio test. Statistical analyses were performed using SAS version 9.4 (SAS Institute Inc., Cary, NC, USA), and a *P* value < 0.05 was considered to indicate significance.

## Results

### Baseline characteristics

In the cohort of 2,077,135 participants, the mean age was 58.3 ± 10.5 years, and 1,176,109 participants (56.6%) were men. The cohort mean lipid profiles are shown in Additional file 1: Table S1. The mean LDL-C and non-HDL-C levels were 113.4 ± 10.5 and 146.3 ± 40.5 mg/dL, respectively. The characteristics of the participants grouped according to LDL-C category are listed in Table 1. Patients in the higher LDL-C categories were more likely to be female and to have a higher fasting glucose level. Patients with a low LDL-C level were more likely to be male, to be current smokers, to be frequent heavy drinkers, and to receive statin medications. The baseline characteristics according to statin use are presented in Additional file 1: Table S2. Statin users were older, more female, and had a lower eGFR and a higher TG level. Statin users had a longer duration of diabetes, were more likely to have hypertension and to receive aspirin.

**Table 1:** Baseline characteristics of subjects according to the low-density lipoprotein cholesterol (LDL-C) levels

	LDL-C1 < 70 mg/dL	LDL-C2 70–99	LDL-C3 100–129	LDL-C4 130–159	LDL-C5 ≥ 160 mg/dL
N	224,131	522,912	630,875	409,247	218,035
Baseline LDL-C (mg/dL)	55.1 ± 11.9	85.9 ± 8.5	114.2 ± 8.6	142.7 ± 8.5	182.1 ± 21.3
Age (years)	59.1 ± 10.3	58.9 ± 10.5	58.3 ± 10.6	57.9 ± 10.6	57.8 ± 10.4
Sex (male)	149,064 (66.5)	323,164 (61.8)	378,583 (60.0)	224,922 (55.0)	100,376 (46.0)
Body mass index (kg/m^2^)	24.8 ± 3.3	24.9 ± 3.3	25.0 ± 3.3	25.2 ± 3.2	25.3 ± 3.2
Systolic BP (mmHg)	128.7 ± 15.6	128.5 ± 15.5	128.9 ± 15.6	129.4 ± 15.7	130.1 ± 16.1
Diastolic BP (mmHg)	78.5 ± 10.1	78.5 ± 10.1	79.0 ± 10.0	79.5 ± 10.1	80.0 ± 10.2
Fasting glucose (mg/dL)	139.1 ± 42.5	140.1 ± 41.6	143.5 ± 41.9	146.9 ± 43.1	151.5 ± 46.4
eGFR (mL/min/1.73 m^2^)	85.9 ± 36.2	85.6 ± 35.5	85.5 ± 34.5	85.1 ± 34.3	84.3 ± 33.8
Baseline TC (mg/dL)	142.1 ± 22.5	168.4 ± 20.1	196.2 ± 19.1	225.3 ± 18.7	266.8 ± 27.9
Baseline HDL-C (mg/dL)	51.7 ± 15.7	51.3 ± 13.6	51.4 ± 12.8	51.8 ± 12.4	53.0 ± 12.4
Baseline TG (mg/dL)	150.5 (150.1–150.8)	136.7 (136.5–136.9)	136.9 (136.7–137.0)	140.0 (139.8–140.2)	146.1 (145.8–146.4)
Current smoker	64,610 (28.8)	132,128 (25.3)	154,612 (24.5)	95,907 (23.4)	46,259 (21.2)
Alcohol drinking	27,933 (12.5)	48,397 (9.3)	51,235 (8.1)	28,894 (7.1)	12,738 (5.8)
Regular exercise	109,775 (49.0)	256,835 (49.1)	307,824 (48.8)	195,661 (47.8)	98,948 (45.4)
Income (lower 25%)	51,468 (23.0)	117,938 (22.6)	142,292 (22.6)	92,628(22.6)	50,908(23.4)
Hypertension	145,727 (65.0)	311,176 (59.5)	346,790 (55.0)	215,537(52.7)	113,059(51.9)
On statin treatment	123,580(55.1)	199,393 (38.1)	144,862 (23.0)	95,709(23.4)	81,496(37.4)
Duration of diabetes (years)	9.2 ± 5.5	8.6 ± 5.7	7.7 ± 5.8	7.1 ± 5.7	6.5 ± 5.4
Use of aspirin	117,987 (52.6)	245,124 (46.9)	254,625 (40.4)	149,627(36.6)	75,142(34.5)

Data are expressed as the mean ± SD, median (25–75%), or n (%)

BP, blood pressure; eGFR, estimated glomerular filtration rate; HDL, high-density lipoprotein; LDL, low-density lipoprotein; TC, total cholesterol; TG, triglyceride

P-values for the trend were < 0.0001 for all variables because of the large size of the study population

### LDL-C level and risk of CVD

**Fig. 1:**
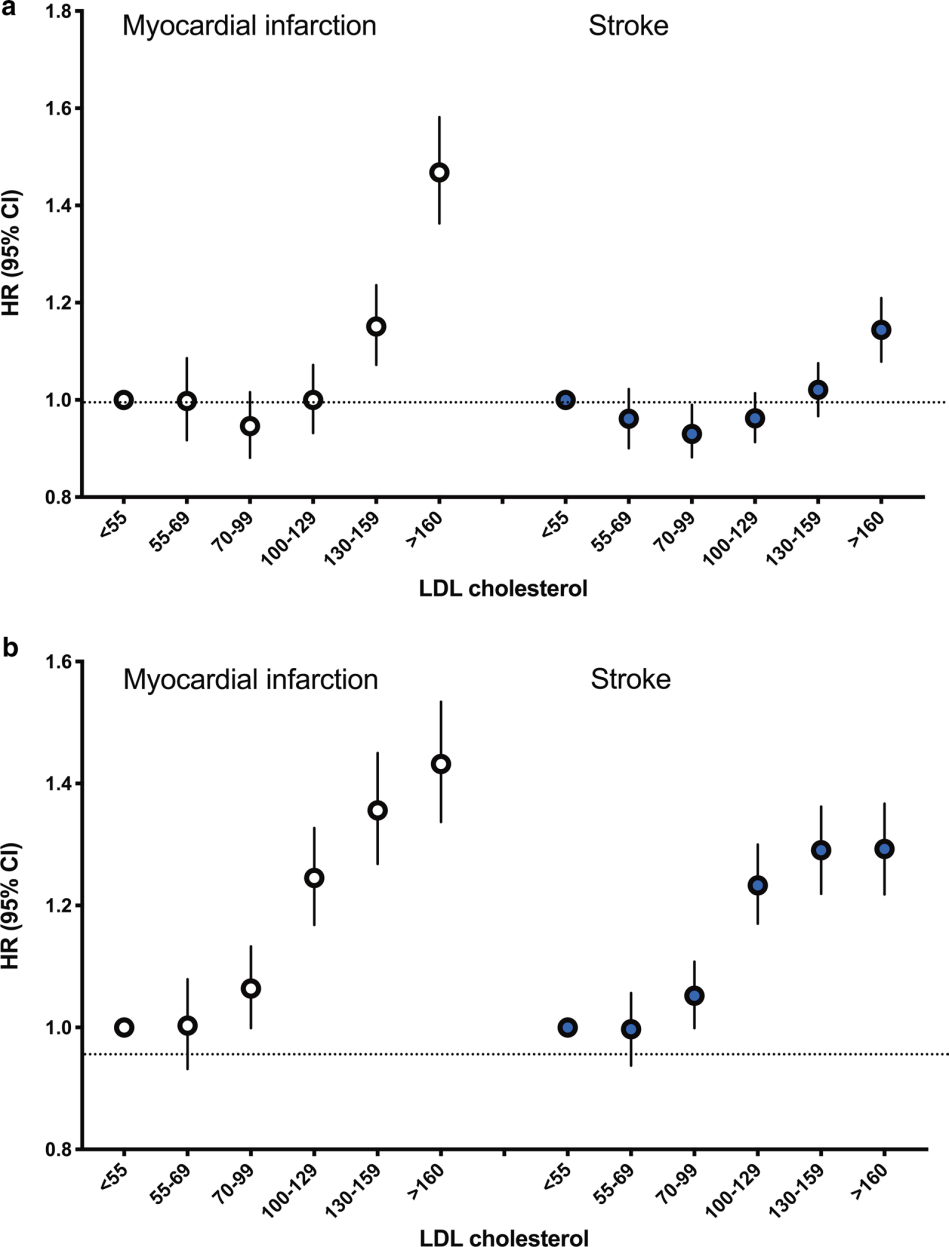
Sensitivity analyses of association between the LDL-C and myocardial infarction, and stroke stratified by statin non-user (**a**) and statin user (**b**); The LDL < 55 mg/dL group was taken as the reference category for the model. Participants were divided into the following categories of LDL-C levels: < 55 mg/dL (reference), 55 to 69 mg/dL, 70 to 99 mg/dL, 100 to 129 mg/dL, 130 to 159 mg/dL, and ≥ 160 mg/dL. Hazard ratios and 95% confidence intervals of myocardial infarction, and stroke according to the low-density lipoprotein cholesterol levels. Adjusted for age, sex, body mass index, alcohol drinking, smoking, regular exercise, income status, fasting glucose levels, hypertension, and duration of diabetes

During a mean 7.1 ± 1.5 years of follow-up, there were 78,560 cases of stroke (3.91%) and 50,791 cases of MI (2.53%). Thirty-two percent of the participants were taking statins. Because statin therapy can affect serum cholesterol levels and outcome incidence, additional analyses were performed by classifying the participants into statin user and non-user groups. Among diabetic participants not taking statins, an LDL-C level ≥ 130 mg/dL was associated with a significantly increased risk of MI and stroke compared with an LDL-C level < 70 mg/dL (Table 2). Using an LDL-C level < 55 mg/dL as the reference, an LDL-C level ≥ 130 mg/dL was associated with a significantly increased risk of MI, and an LDL-C level ≥ 160 mg/dL was associated with an increased risk of stroke (Fig. 1a).

**Table 2:** Risk of myocardial infarction and stroke in patients with type 2 diabetes mellitus according to low-density lipoprotein cholesterol (LDL-C) category

	Total			Statin non-user			Statin user		
	Events (n)	Incidence rate (per 1000 person-years)	HR (95% CI)*	Events (n)	Incidence rate (per 1000 person-years)	HR (95% CI)*	Events (n)	Incidence rate (per 1000 person-years)	HR (95% CI)*
Myocardial infarction									
< 70	5342	3.50	1 (ref.)	2330	3.44	1 (ref.)	3012	3.55	1 (ref.)
70–99	12,274	3.41	1.02 (0.99, 1.06)	7152	3.22	0.95 (0.91, 1.00)	5122	3.71	1.07 (1.03, 1.12)
100–129	15,366	3.52	1.12 (1.09, 1.16)	11,122	3.31	1.02 (0.97, 1.07)	4244	4.22	1.26 (1.20, 1.32)
130–159	10,981	3.88	1.29 (1.25, 1.34)	7985	3.69	1.19 (1.14, 1.25)	2996	4.51	1.39 (1.32, 1.46)
≥ 160	6828	4.56	1.56 (1.51, 1.62)	4228	4.53	1.53 (1.46, 1.62)	2600	4.60	1.49 (1.41, 1.57)
P for trend			< 0.0001			< 0.0001			< 0.0001
Stroke									
< 70	8527	5.63	1 (ref.)	4077	6.07	1 (ref.)	4450	5.27	1 (ref.)
70–99	19,873	5.56	1.03 (1.00, 1.05)	12,387	5.62	0.96 (0.93, 1.00)	7486	5.46	1.07 (1.03, 1.11)
100–129	24,759	5.72	1.11 (1.08, 1.14)	18,611	5.59	1.01 (0.97, 1.04)	6148	6.16	1.25 (1.20, 1.30)
130–159	16,354	5.82	1.19 (1.15, 1.22)	12,211	5.68	1.08 (1.04, 1.12)	4143	6.27	1.31 (1.26, 1.37)
≥ 160	9047	6.07	1.30 (1.26, 1.34)	5655	6.09	1.21 (1.17, 1.26)	3392	6.04	1.33 (1.27, 1.40)
P for trend			< 0.0001			< 0.0001			< 0.0001

HR, hazard ratio; CI, confidence interval

* Adjusted for age, sex, BMI, smoking, alcohol drinking, exercise, income status, use of statins, fasting glucose levels, hypertension, and duration of diabetes

The risk of MI and stroke increased among diabetic people with type 2 DM having an on-treatment LDL-C level ≥ 70 mg/dL (Table 2). The multivariable-adjusted hazard ratios (HRs) for MI and stroke increased linearly from an LDL-C level ≥ 70 mg/dL. LDL-C levels 70–99 mg/dL and 100–129 mg/dL were associated with a significant increase in the incidence of MI; the HRs were 1.07 (95% confidence interval [CI] 1.03–1.12, *P* < 0.001) and 1.26 (95% CI 1.20–1.32, *P* < 0.001), respectively, compared with an LDL-C level < 70 mg/dL. In the analysis with LDL-C < 55 mg/dL as the reference, an LDL-C level 55–69 mg/dL was not associated with an increased risk of CVD, but an LDL-C level ≥ 70 mg/dL was associated with a significant increase in the incidence of CVD (Fig. 1b).

Participants were classified into five groups according to LDL-C quintile (Additional file 1: Table S3). The risk of MI among diabetic people not taking statins significantly increased in the fourth quintile (LDL-C: 121–142 mg/dL; HR: 1.13, 95% CI 1.09–1.17) and fifth quintile (LDL-C: ≥ 143 mg/dL; HR: 1.41, 95% CI 1.36–1.47) compared with the first quintile of LDL-C level (< 81 mg/dL). By contrast, the risk of stroke increased only in the fifth quintile (LDL-C: ≥ 143 mg/dL; HR: 1.17, 95% CI 1.14–1.21). The risk of MI or stroke among diabetic patients receiving statin therapy increased linearly from the second quintile of LDL-C (82–102 mg/dL).

### Non-HDL-C level and risk of CVD

Among participants not taking statins, the non-HDL-C categories 130–159 mg/dL, 160–189 mg/dL, and ≥ 190 mg/dL continued to be significantly associated with the risk of MI; the HRs (95% CI) were 1.07 (1.02–1.12), 1.26 (1.20–1.31), and 1.64 (1.57–1.72), respectively (Table 3). The non-HDL-C category ≥ 160 mg/dL was significantly associated with an increased risk of stroke in multivariable models. Among participants taking statins, all categories of non-HDL-C ≥ 100 mg/dL were significantly associated with an increased risk of stroke and MI. Similar results were obtained when classifying the participants into five groups according to non-HDL-C quintiles (Additional file 1: Table S4).

**Table 3:** Risk of myocardial infarction and stroke in patients with type 2 diabetes mellitus according to non-high-density lipoprotein cholesterol (non-HDL-C) category

	Total			Statin non-user			Statin user		
	Events (n)	Incidence rate (per 1000 person-years)	HR (95% CI)*	Events (n)	Incidence rate (per 1000 person-years)	HR (95% CI)*	Events (n)	Incidence rate (per 1000 person-years)	HR (95% CI)*
Myocardial infarction									
< 100	5609	3.37	1 (ref.)	2521	3.38	1 (ref.)	3088	3.36	1 (ref.)
100–129	11,540	3.35	1.07 (1.03, 1.10)	6898	3.13	0.97 (0.92, 1.0)	4642	3.76	1.16 (1.11, 1.21)
130–159	14,768	3.51	1.19 (1.15, 1.23)	10,638	3.30	1.07 (1.02, 1.12)	4130	4.18	1.33 (1.27, 1.40)
160–189	11,614	3.87	1.38 (1.34, 1.43)	8248	3.66	1.26 (1.20, 1.31)	3366	4.49	1.49 (1.42, 1.57)
≥ 190	9158	4.57	1.69 (1.63, 1.75)	5607	4.50	1.64 (1.57, 1.72)	3551	4.68	1.63 (1.55, 1.71)
P for trend			< 0.0001			< 0.0001			< 0.0001
Stroke									
< 100	9171	5.55	1 (ref.)	4514	6.10	1 (ref.)	4657	5.10	1 (ref.)
100–129	18,768	5.49	1.05 (1.02, 1.07)	12,045	5.50	0.96 (0.92, 0.99)	6723	5.47	1.13 (1.08, 1.17)
130–159	23,725	5.68	1.15 (1.12, 1.17)	17,766	5.56	1.03 (0.99, 1.06)	5959	6.07	1.30 (1.25, 1.35)
160–189	17,435	5.85	1.25 (1.22, 1.29)	12,748	5.70	1.12 (1.08, 1.16)	4687	6.30	1.42 (1.36, 1.48)
≥ 190	12,194	6.11	1.39 (1.35, 1.43)	7563	6.10	1.29 (1.24, 1.34)	4631	6.14	1.45 (1.39, 1.52)
P for trend			< 0.0001			< 0.0001			< 0.0001

HR, hazard ratio; CI, confidence interval

* Adjusted for age, sex, BMI, smoking, alcohol drinking, exercise, income status, use of statins, fasting glucose levels, hypertension, and duration of diabetes

### Risk of CVD according to LDL-C category in subgroups according to age and sex

Next, the associations between LDL-C level and incident CVD were examined in subgroups of the study participants. The interaction between LDL-C level and age in the development of MI was significant (*P* for interaction < 0.001). The association between LDL-C level and MI risk was stronger in younger than in older participants. Among diabetic participants not taking statins, an LDL-C level ≥ 130 mg/dL was associated with a significantly increased risk of MI and stroke in both young and old populations. The interaction between LDL-C level and sex in the development of MI was also significant (*P* for interaction < 0.005). The association between LDL-C level and MI/stroke risk was stronger in men than in women Additional file 1: Table S5). Among diabetic women not taking statins, an LDL-C level ≥ 160 mg/dL was associated with a significantly increased risk of MI. In men, the risk of MI began to increase at LDL-C ≥ 100 mg/dL.

To account for the possibility of reverse causation, a sensitivity analysis was performed by excluding participants with the occurrence of end points within 2 years of follow-up. The results were similar to those of the original analysis (Table 4). The pooled outcome analysis showed similar results to those previously analyzed separately for MI or stroke (Additional file 1: Table S6).

**Table 4:** Hazard ratios and 95% confidence intervals of myocardial infarction and stroke by low-density lipoprotein cholesterol (LDL-C) category; Sensitivity analysis excluding subjects with the occurrence of end points within 2 year of follow-up

	Total			Statin non-user			Statin user		
	Events (n)	Incidence rate (per 1000 person-years)	HR (95% CI)*	Events (n)	Incidence rate (per 1000 person-years)	HR (95% CI)*	Events (n)	Incidence rate (per 1000 person-years)	HR (95% CI)*
MI									
< 70	4058	3.78	1 (ref.)	1788	3.77	1 (ref.)	2270	3.79	1 (ref.)
70–99	9274	3.65	1.00 (0.96, 1.03)	5467	3.49	0.94 (0.90, 1.00)	3807	3.90	1.05 (1.00, 1.11)
100–129	11,518	3.74	1.07 (1.03, 1.11)	8591	3.62	1.02 (0.97, 1.07)	2927	4.12	1.15 (1.09, 1.21)
130–159	8182	4.10	1.22 (1.18, 1.27)	6209	4.06	1.20 (1.13, 1.26)	1973	4.20	1.21 (1.14, 1.29)
≥ 160	4887	4.63	1.44 (1.38, 1.50)	3148	4.80	1.48 (1.40, 1.57)	1739	4.36	1.32 (1.24, 1.41)
P for trend			< 0.0001			< 0.0001			< 0.0001
Stroke									
< 70	6434	6.03	1 (ref.)	3083	6.54	1 (ref.)	3351	5.62	1 (ref.)
70–99	14,892	5.89	1.00 (0.97, 1.03)	9410	6.04	0.96 (0.92, 1.01)	5482	5.64	1.03 (0.99, 1.08)
100–129	18,137	5.91	1.04 (1.01, 1.07)	14,011	5.94	0.99 (0.95, 1.03)	4126	5.83	1.11 (1.06, 1.16)
130–159	11,707	5.89	1.08 (1.05, 1.12)	9052	5.95	1.05 (1.01, 1.09)	2655	5.68	1.12 (1.07, 1.18)
≥ 160	6280	5.97	1.17 (1.13, 1.21)	4070	6.22	1.15 (1.09, 1.20)	2210	5.56	1.16 (1.11, 1.23)
P for trend			< 0.0001			< 0.0001			< 0.0001

HR, hazard ratio; CI, confidence interval

* Adjusted for age, sex, BMI, smoking, alcohol drinking, exercise, income status, use of statins, fasting glucose levels, hypertension, and duration of diabetes

## Discussion

In this large population-based prospective study of Koreans with type 2 DM, significant positive associations were observed between an increased risk of CVD and high LDL-C and non-HDL-C levels. We identified an increased risk of CVD with an LDL-C level ≥ 130 mg dL in patients with type 2 DM who were not taking statins. The risk of CVD was significantly increased in the statin-treated participants with an LDL-C level ≥ 70 mg/dL.

It is clear that people with low cholesterol levels gain less absolute benefit from cholesterol-lowering therapy than do people with high cholesterol levels [13]. For the present study population, the mean LDL-C and non-HDL cholesterol levels at baseline were 113 mg/dL and 146 mg/dL, respectively. Previous studies involving participants with higher mean LDL-C levels showed an association with the risk of coronary heart disease (CHD) for those in the higher LDL-C ranges. These studies include the Framingham study, in which the mean LDL-C levels at baseline were 139 mg/dL for men and 138 mg/dL for women [14]. In a cholesterol-lowering clinical trial of intermediate-risk patients (Heart Outcomes Prevention Evaluation-3 [HOPE-3]), the pretreatment LDL-C level was 129 mg/dL [15]. The goals and starting points of cholesterol treatment may be different in these low-cholesterol population. However, indication for starting statin therapy should also be related to the progression of atherosclerosis or clinical event regardless on a level of baseline cholesterol [16–18]. For the prediction of MI or stroke, the area under the curve of LDL-C alone was low (data not shown), suggesting that including other potential risk factors such as smoking, BP, or family history of premature vascular disease to the risk model would be beneficial for a better risk prediction of CVD rather than LDL-C alone [18, 19]. Statins might be employed in patients without CVD but with elevated cholesterol and/or multiple atherosclerotic risk factors. In this study, we aimed to determine the level of “elevated cholesterol” in which statin therapy should be started in patients without CVD. This study suggested that statin therapy should be considered when LDL-C was above 130 mg/dL, regardless of CVD risk factors in diabetic patients without CVD. In this study, previous use of statin was dependent on the clinical judgement of the physicians. Statin users tended to have other cardiovascular risk factors, including hypertension and longer diabetes duration. According to the European Society of Cardiology guidelines [20], diabetic patients at high risk should be intensively treated with statin therapy with an LDL-C goal of < 70 mg/dl. Our results are consistent with the recommendation; The risk of MI and stroke increased among diabetic people with type 2 DM having an on-treatment LDL-C level ≥ 70 mg/dL.

A previous Japanese population-based cohort study showed a nonlinear association. The multivariable HR for CHD was 1.68-times higher for people with an LDL-C level 126–150 mg/dL compared with men and women whose level was ≤ 102 mg/dL, and the risk plateaued at < 125 mg/dL [21]. Another Japanese cohort study showed that the mean LDL-C levels were 110.5 mg/dL for men [13]. In that study, men with an LDL-C level ≥ 140 mg/dL had twofold higher age-adjusted risk of mortality for CHD than did those with an LDL-C level < 80 mg/dL [13]. In Chinese patients with type 2 DM without a history of CVD and no lipid-modifying drug use, the HR for CVD increased sharply when the LDL-C level was > 116 mg/dL [22]. We observed a graded positive trend for MI risk starting from an LDL-C level of 130 mg/dL, which increased for the higher LDL-C categories among people with type 2 DM who were not taking statins. Our data suggests that statin therapy might be considered for people whose LDL-C level remains ≥ 130 mg/dL after appropriate lifestyle modifications.

In the present study, an association between non-HDL-C level and MI risk was seen at a lower level of non-HDL-C than for the corresponding LDL-C level. In a recent study of relatively low-risk people without a history of atherosclerotic CVD (ASCVD) or DM at baseline, the association between LDL-C (or non-HDL-C) levels and ASCVD mortality was evaluated over a median follow-up of 27 years [23]. Compared with participants with an LDL-C level < 100 mg/dL, those with an LDL-C level ≥ 160 mg/dL had an increased risk for CVD mortality. In that study, a non-HDL-C level ≥ 160 mg/dL was significantly associated with CVD death compared with a non-HDL-C level < 130 mg/dL [23]; our findings are consistent with the results of that study. We also found that, among diabetic people not taking statins, LDL-C and non-HDL-C levels ≥ 130 mg/dL were independently associated with a significant increase in the incidence of MI. The non-HDL-C level captures the risk associated with both cholesterol-rich lipoproteins and TG-rich remnant lipoproteins [24]. We note that all recent outcomes trials of proprotein convertase subtilisin/kexin type 9 inhibitors used both LDL-C and non-HDL-C thresholds for deciding eligibility, and may therefore have included people with an elevated non-HDL-C level despite having an LDL-C level below the eligibility criterion [25].

In the present study, the risk of CVD was higher in participants whose LDL-C level was greater than the on-treatment level of > 70 mg/dL; however, no increase in risk was observed for those whose LDL-C level was in the range 55–69 mg/dL. It was recently reported that the intensive statin therapy target LDL-C level of < 70 mg/dL did not reduce the incidence of composite CV events more significantly than the standard therapy target LDL-C level of ≥ 100 and < 120 mg/dL [6]. However, in that study, targeted levels were achieved in < 50% of patients. When analyzed the data from 1909 patients who achieved the target LDL-C level, achieving an LDL-C level < 70 mg/dL was associated with a more effective reduction in CV events incidence than the target level of 100 to 120 mg/dL in patients with type 2 DM with retinopathy [26].

Application of the American Heart Association–American College of Cardiology–atherosclerotic cardiovascular disease (AHA-ACC-ASCVD) score overestimated cardiovascular events in a multiethnic cohort, including Chinese people, without baseline clinical ASCVD [27]. The Framingham risk score was developed based on data collected from the Caucasian population and overestimates the 10-year risk of CHD in the Asian population [28, 29]. A study using data from the Hong Kong Diabetes Registry, established in 1995, also reported that the United Kingdom Prospective Diabetes Study Risk Engine overestimates the risk of CVD with suboptimal discrimination [30]. There is significant heterogeneity in CVD prevalence between populations with diabetes. These discrepancies in CVD prevalence may be related to differences in disease profile and other determinants such as genetics, health care policy, and culture. A population-based CVD risk prediction model for Asian people with type 2 DM should be developed to allow for better management. The thresholds for initiating treatment of abnormal lipid levels and the optimal goals of lipid-altering therapy may also differ according to ethnicity or population [31–33].

The optimal LDL-C threshold for starting statin therapy may also vary by age and sex [18, 34]. The association between LDL-C and MI risk in elderly patients was weak. Lipid levels measured in late life may decrease as a result of behavioral changes or because of the presence of comorbidities.

The association between LDL-C and risk of MI was stronger in men than in women, suggesting that there are more atherogenic characteristics of LDL-C in men. It was also reported that risk of CHD was associated with high TG and low HDL-C in women and high LDL-C in men [35]. There has been controversy over whether statins are effective for primary prevention in women [20]. The gender gap in risk of CHD persists throughout life but the relative risk declines with age [36]. What causes the gender contrast in risk is still not clear. An adverse effect of male hormones, gender heterogeneity in insulin resistance mechanisms, in LDL-C characteristics and/or in aging processes influencing arterial stiffness, are some of the theories suggested [36]. The epidemiological evidence for an association of high LDL-C with an increased risk of ischemic stroke is relatively weak [21]. We also found that a stroke was less related to the cholesterol level than MI. It may be due to the heterogenous etiology of stroke. Lacunar infarction and cardioembolic infarction seem to have a less potent relation with elevated LDL-C [21]. Although it was difficult to clearly define the subtypes of stroke, we excluded hemorrhagic stroke (ICD-10 codes I60, I61, I62). We provided cut-offs for the LDL-C levels (e.g. 70, 100, 130 mg/dL), which were different from the observed quintiles. In major guidelines on the treatment of blood cholesterol to reduce CVD [20, 37], LDL-C goals < 55, 70, 100, or 130 mg/dL are recommended depending to the CV risk.

There are several limitations to our study. First, day-to-day variability because of laboratory error or biological variability may have affected the results because we used a single measurement of cholesterol levels. Repeated measurement would have been more accurate for classifying the participants. Second, several studies [38, 39] have shown that even when the TG level is < 400 mg/dL, a higher TG level can increase the degree to which the Friedewald formula underestimates LDL-C level. We excluded participants with a TG level > 400 mg/dL, but we also analyzed non-HDL cholesterol levels. Third, because the analysis was performed using an NHIS health examinee cohort, diabetic patients with severe disabilities would likely have been excluded from undergoing regular health examinations. Because patients with underlying CVD at baseline were excluded, the study population likely included mostly relatively low-risk diabetic participants. Fourth, cholesterol levels may increase over time and trigger the use of statins among non-users, such that the observed risk of CVD would be mitigated. Our study was limited in that time-varying Cox regression was not performed. Fifth, we were unable to obtain clinical information on hemoglobin A1c levels, high sensitivity C-reactive protein [40], and other medications that an affect the development of CVD. Lastly, because only the Korean population was included, our findings cannot be extrapolated to people with different ethnicities. Despite these limitations, this study evaluated longitudinal data from the entire Korean adult population. Therefore, our findings reflect ‘real-world’ data, on a national scale, regarding the effects of LDL-C and non-HDL-C levels on CVD risk in people with type 2 DM.

## Conclusion

Although the existence of a threshold does not always mean that the optimal cutoff level should be the same value, the absence of an increase in risk below this threshold suggests that the LDL-C cutoff point for Korean people with DM that increases the risk of CVD may be around 130 mg/dL. Because we observed a graded positive trend in MI risk starting from a LDL-C level of 130 mg/dL, and this risk increased for the higher LDL-C categories, we recommend initiating statin treatment for primary prevention of CVD in Korean patients with type 2 DM when the LDL-C level is ≥ 130 mg/dL. We found a significantly increased risk of CVD in statin-treated participants with an LDL-C level ≥ 70 mg/dL. For patients with type 2 DM without preexisting CVD, our recommendations for starting statin therapy for primary prevention of CVD are: 1) an LDL cholesterol level ≥ 130 mg/dL, and 2) a goal LDL-C level of < 70 mg/dL.

## Supplementary information

**Additional file 1.** Additional Tables and Figure.

## Data Availability

The datasets used and/or analyzed in the current study are available from the corresponding author upon reasonable request.

## Abbreviations

ASCVD: atherosclerotic cardiovascular disease
BP: blood pressure
CHD: coronary heart disease
CVD: cardiovascular disease
DM: diabetes mellitus
HDL-C: high-density cholesterol
ICD: International Classification of Diseases
LDL-C: low-density lipoprotein cholesterol
MI: myocardial infarctions
NHIS: National Health Insurance System
TG: triglyceride
