# Supplementary material for: Cholesterol levels and development of cardiovascular disease in Koreans with type 2 diabetes mellitus and without pre-existing cardiovascular disease
Source: Cardiovasc Diabetol. 2019 Oct 22;18:139. doi: 10.1186/s12933-019-0943-9 (PMC6805335; doi:10.1186/s12933-019-0943-9)
Supplement: Supplementary file 1 — Additional file 1. Additional Tables and Figure. [file 12933_2019_943_MOESM1_ESM.doc]

Table S1. The mean lipid profile levels of the study population

|  | Total population |
| --- | --- |
| Age (years) | 58.3 ± 10.5 |
| Total cholesterol (mg/dL) | 197.7 ± 41.5 |
| HDL cholesterol (mg/dL) | 51.4 ± 13.3 |
| LDL cholesterol (mg/dL) | 113.4 ± 10.5 |
| Non-HDL cholesterol (mg/dL) | 146.3 ± 40.5 |
| Triglyceride (mg/dL) | 146.5 (146.4, 146.6) |

Data are expressed as the means ± SD or median (25-75%). HDL, high density lipoprotein; LDL, low-density lipoprotein

Table S2. Comparison of the baseline characteristics of the subjects according to the statin use.

|  | Statin Non-user  (n=1,405,748) | Statin User  (n=671,387) |
| --- | --- | --- |
| Age (years) | 57.7±10.8 | 59.5±9.8 |
| Sex (male) | 897133(63.8) | 335430(50.0) |
| Body mass index (kg/m^2^) | 24.8±3.3 | 25.5±3.2 |
| Systolic BP (mmHg) | 129.2±15.8 | 128.9±15.5 |
| Diastolic BP (mmHg) | 79.5±10.2 | 78.5±10.0 |
| Fasting glucose (mg/dL) | 146.5±43.0 | 139.9±43.7 |
| eGFR (ml/min/1.73 m^2^) | 86.3±34.8 | 83.5±34.9 |
| Baseline TC (mg/dL) | 200.2±37.9 | 192.4±47.8 |
| Baseline HDL-C (mg/dL) | 51.4±13.4 | 51.5±13.1 |
| Baseline TG (mg/dL) | 144.0(143.9-144.1) | 151.8(151.6-152.0) |
| Current smoker | 386905(27.5) | 137427(20.5) |
| Alcohol drinking | 135737(9.7) | 47677(7.1) |
| Regular exercise | 683490(48.6) | 321509(47.9) |
| Income (lower 25%) | 322610(23.0) | 149077(22.2) |
| Hypertension | 717171(51.0) | 456630(68.0) |
| Duration of diabetes (years) | 6.9±5.8 | 9.7±5.0 |
| Use of aspirin | 483295(34.4) | 382951(57.0) |

Data are expressed as the means ± SD, median (25-75%), or n (%).

P-values for the trend were < 0.0001 for all variables because of the large size of the study population.

BP, blood pressure; eGFR, estimated glomerular filtration rate; HDL, high-density lipoprotein; LDL, low-density lipoprotein; TC, total cholesterol; TG, triglyceride

Table S3. Risk of myocardial infarction and stroke in patients with type 2 diabetes mellitus according to quintiles of low-density lipoprotein (LDL) cholesterol

|  | Total |  | |  | Statin Non-user | | | Statin User | | |
| --- | --- | --- | --- | --- | --- | --- | --- | --- | --- | --- |
|  | Events (n) | Incidence rate (per 1000 person-years) | HR (95% CI)* | | Events (n) | Incidence rate (per 1000 person-years) | HR (95% CI)* | Events (n) | Incidence rate (per 1000 person-years) | HR (95% CI)* |
| MI |  |  |  | |  |  |  |  |  |  |
| Q1(-81) | 9349 | 3.44 | 1(ref.) | | 4435 | 3.36 | 1(ref.) | 4914 | 3.51 | 1(ref.) |
| Q2(82-101) | 9843 | 3.46 | 1.06(1.03,1.09) | | 6119 | 3.23 | 0.98(0.94,1.02) | 3724 | 3.92 | 1.15(1.10,1.20) |
| Q3(102-120) | 9402 | 3.49 | 1.12(1.09,1.15) | | 6758 | 3.28 | 1.03(0.99,1.06) | 2644 | 4.19 | 1.26(1.20,1.32) |
| Q4(121-142) | 10400 | 3.71 | 1.23(1.20,1.27) | | 7745 | 3.50 | 1.13(1.09,1.17) | 2655 | 4.47 | 1.37(1.31,1.44) |
| Q5(143-) | 11797 | 4.29 | 1.47(1.43,1.51) | | 7760 | 4.17 | 1.41(1.36,1.47) | 4037 | 4.54 | 1.45(1.39,1.51) |
| P for trend |  |  | <0.0001 | |  |  | <0.0001 |  |  | <0.0001 |
| Stroke |  |  |  | |  |  |  |  |  |  |
| Q1(-81) | 15210 | 5.63 | 1(ref.) | | 7804 | 5.96 | 1(ref.) | 7406 | 5.32 | 1(ref.) |
| Q2(82-102) | 15721 | 5.56 | 1.03(1.01,1.05) | | 10467 | 5.56 | 0.97(0.94,1.00) | 5254 | 5.56 | 1.08(1.04,1.12) |
| Q3(102-120) | 15257 | 5.71 | 1.09(1.07,1.12) | | 11427 | 5.59 | 1.01(0.98,1.04) | 3830 | 6.11 | 1.22(1.17,1.27) |
| Q4(121-142) | 15994 | 5.74 | 1.14(1.11,1.17) | | 12220 | 5.57 | 1.04(1.01,1.07) | 3774 | 6.41 | 1.31(1.26,1.36) |
| Q5(143-) | 16378 | 5.99 | 1.25(1.22,1.27) | | 11023 | 5.96 | 1.17(1.14,1.21) | 5355 | 6.06 | 1.29(1.25,1.34) |
| P for trend |  |  | <0.0001 | |  |  | <0.0001 |  |  | <0.0001 |

HR, hazard ratio; CI, confidence interval

*Adjusted for age, sex, BMI, smoking, alcohol drinking, exercise, income status, use of statins, fasting glucose levels, hypertension, and duration of diabetes

Table S4. Risk of myocardial infarction and stroke in patients with type 2 diabetes mellitus according to quintiles of non-high-density lipoprotein cholesterol (Non-HDL-C)

|  | Total |  | |  | Statin Non-user | | | Statin User | | |
| --- | --- | --- | --- | --- | --- | --- | --- | --- | --- | --- |
|  | Events (n) | Incidence rate (per 1000 person-years) | HR (95% CI)* | | Events (n) | Incidence rate (per 1000 person-years) | HR (95% CI)* | Events (n) | Incidence rate (per 1000 person-years) | HR (95% CI)* |
| MI |  |  |  | |  |  |  |  |  |  |
| Q1(-111) | 9440 | 3.34 | 1(ref.) | | 4626 | 3.27 | 1(ref.) | 4814 | 3.41 | 1(ref.) |
| Q2(112-133) | 9695 | 3.41 | 1.10(1.06,1.13) | | 6137 | 3.14 | 1.01(0.96,1.04) | 3558 | 3.98 | 1.22(1.16,1.27) |
| Q3(134-153) | 9879 | 3.47 | 1.17(1.13,1.20) | | 7145 | 3.27 | 1.08(1.04,1.12) | 2734 | 4.13 | 1.29(1.23,1.35) |
| Q4(154-178) | 10915 | 3.76 | 1.32(1.28,1.35) | | 7924 | 3.55 | 1.22(1.18,1.27) | 2991 | 4.47 | 1.44(1.37,1.51) |
| Q5(179-) | 12760 | 4.40 | 1.60(1.56,1.64) | | 8080 | 4.28 | 1.57(1.51,1.63) | 4680 | 4.63 | 1.56(1.50,1.63) |
| P for trend |  |  | <0.0001 | |  |  | <0.0001 |  |  | <0.0001 |
| Stroke |  |  |  | |  |  |  |  |  |  |
| Q1(-111) | 15376 | 5.47 | 1(ref.) | | 8152 | 5.81 | 1(ref.) | 7224 | 5.14 | 1(ref.) |
| Q2(112-133) | 15630 | 5.53 | 1.07(1.04,1.09) | | 10633 | 5.49 | 1.00(0.97,1.03) | 4997 | 5.63 | 1.15(1.11,1.19) |
| Q3(134-153) | 16189 | 5.73 | 1.15(1.13,1.18) | | 12113 | 5.59 | 1.06(1.03,1.09) | 4076 | 6.19 | 1.31(1.26,1.36) |
| Q4(154-178) | 16706 | 5.80 | 1.22(1.20,1.25) | | 12500 | 5.64 | 1.13(1.09,1.16) | 4206 | 6.33 | 1.38(1.33,1.44) |
| Q5(179-) | 17392 | 6.03 | 1.35(1.32,1.39) | | 11238 | 5.99 | 1.28(1.24,1.32) | 6154 | 6.12 | 1.41(1.36,1.46) |
| P for trend |  |  | <0.0001 | |  |  | <0.0001 |  |  | <0.0001 |

HR, hazard ratio; CI, confidence interval

*Adjusted for age, sex, BMI, smoking, alcohol drinking, exercise, income status, use of statins, fasting glucose levels, hypertension, and duration of diabetes

Table S5. Subgroup analyses of association between the low-density lipoprotein cholesterol (LDL-C) and stroke and myocardial infarction stratified by age and sex.

| Subgroup | LDL-C | Myocardial infarction | | | | Stroke | | | |
| --- | --- | --- | --- | --- | --- | --- | --- | --- | --- |
|  |  | Statin Non-user | | Statin User | | Statin Non-user | | Statin User | |
| Age |  | HR (95% CI)* | P for interaction | HR (95% CI)* | P for interaction | HR (95% CI)* | P for interaction | HR (95% CI)* | P for interaction |
| 40-64 years | <70 | 1(ref.) | <0.001 | 1(ref.) | 0.1689 | 1(ref.) | <0.001 | 1(ref.) | 0.05 |
|  | 70-99 | 0.90(0.84,0.96) |  | 1.10(1.03,1.17) |  | 0.95(0.90,1.00) |  | 1.05(0.99,1.11) |  |
|  | 100-129 | 1.01(0.95,1.08) |  | 1.31(1.22,1.40) |  | 0.97(0.92,1.02) |  | 1.31(1.24,1.40) |  |
|  | 130-159 | 1.23(1.15,1.31) |  | 1.45(1.35,1.56) |  | 1.07(1.01,1.13) |  | 1.37(1.29,1.47) |  |
|  | ≥160 | 1.68(1.56,1.80) |  | 1.68(1.56,1.81) |  | 1.28(1.21,1.36) |  | 1.37(1.27,1.47) |  |
| ≥65 years | <70 | 1(ref.) |  | 1(ref.) |  | 1(ref.) |  | 1(ref.) |  |
|  | 70-99 | 1.01(0.94,1.07) |  | 1.05(0.98,1.12) |  | 0.98(0.94,1.03) |  | 1.07(1.02,1.13) |  |
|  | 100-129 | 1.02(0.96,1.09) |  | 1.21(1.14,1.30) |  | 1.04(0.99,1.09) |  | 1.21(1.14,1.27) |  |
|  | 130-159 | 1.14(1.07,1.22) |  | 1.33(1.23,1.43) |  | 1.10(1.05,1.15) |  | 1.27(1.20,1.35) |  |
|  | ≥160 | 1.38(1.28,1.48) |  | 1.32(1.22,1.43) |  | 1.18(1.11,1.24) |  | 1.32(1.24,1.40) |  |
| Sex |  | HR (95% CI)* | P for interaction | HR (95% CI)* | P for interaction | HR (95% CI)* | P for interaction | HR (95% CI)* | P for interaction |
| Men | <70 | 1(ref.) | <0.001 | 1(ref.) | <0.001 | 1(ref.) | <0.001 | 1(ref.) | 0.005 |
|  | 70-99 | 0.95(0.90,1.01) |  | 1.10(1.03,1.16) |  | 0.96(0.92,1.00) |  | 1.07(1.02,1.12) |  |
|  | 100-129 | 1.07(1.02,1.13) |  | 1.33(1.25,1.40) |  | 1.01(0.97,1.05) |  | 1.32(1.26,1.39) |  |
|  | 130-159 | 1.27(1.20,1.34) |  | 1.46(1.36,1.57) |  | 1.11(1.06,1.16) |  | 1.34(1.26,1.42) |  |
|  | ≥160 | 1.76(1.66,1.88) |  | 1.72(1.60,1.85) |  | 1.30(1.23,1.37) |  | 1.39(1.30,1.48) |  |
| Women | <70 | 1(ref.) |  | 1(ref.) |  | 1(ref.) |  | 1(ref.) |  |
|  | 70-99 | 0.93(0.85,1.01) |  | 1.02(0.96,1.10) |  | 0.99(0.92,1.06) |  | 1.05(0.99,1.11) |  |
|  | 100-129 | 0.88(0.81,0.96) |  | 1.16(1.08,1.24) |  | 1.01(0.94,1.08) |  | 1.17(1.10,1.24) |  |
|  | 130-159 | 1.01(0.92,1.10) |  | 1.27(1.18,1.38) |  | 1.05(0.98,1.12) |  | 1.27(1.20,1.35) |  |
|  | ≥160 | 1.20(1.09,1.31) |  | 1.28(1.18,1.38) |  | 1.14(1.06,1.23) |  | 1.28(1.19,1.36) |  |

HR, hazard ratio; CI, confidence interval

*Adjusted for age, sex, BMI, smoking, alcohol drinking, exercise, income status, use of statins, fasting glucose levels, hypertension, and duration of diabetes

Table S6. Risk of the pooled outcome (combining myocardial infarction or stroke) in patients with type 2 diabetes mellitus according to low-density lipoprotein cholesterol (LDL-C) or non-high-density lipoprotein cholesterol (Non-HDL-C) category

|  | Total |  | |  | Statin Non-user | | | Statin User | | |
| --- | --- | --- | --- | --- | --- | --- | --- | --- | --- | --- |
| LDL-C | Events (n) | Incidence rate (per 1000 person-years) | HR (95% CI)* | | Events (n) | Incidence rate (per 1000 person-years) | HR (95% CI)* | Events (n) | Incidence rate (per 1000 person-years) | HR (95% CI)* |
| <70 | 13162 | 8.76 | 1(ref.) | | 6111 | 9.17 | 1(ref.) | 7051 | 8.43 | 1(ref.) |
| 70-99 | 30554 | 8.62 | 1.03(1.01,1.05) | | 18565 | 8.49 | 0.96(0.93,0.98) | 11989 | 8.82 | 1.08(1.05,1.11) |
| 100-129 | 38034 | 8.86 | 1.12(1.10,1.14) | | 28228 | 8.54 | 1.01(0.98,1.04) | 9806 | 9.94 | 1.26(1.22,1.30) |
| 130-159 | 25962 | 9.34 | 1.23(1.21,1.26) | | 19189 | 9.01 | 1.12(1.09,1.15) | 6773 | 10.40 | 1.36(1.32,1.41) |
| ≥160 | 15021 | 10.21 | 1.41(1.37,1.44) | | 9354 | 10.20 | 1.33(1.29,1.38) | 5667 | 10.23 | 1.41(1.36,1.46) |
| P for trend |  |  | <0.0001 | |  |  | <0.0001 |  |  | <0.0001 |
| Non-HDL-C | Events (n) | Incidence rate (per 1000 person-years) | HR (95% CI)* | | Events (n) | Incidence rate (per 1000 person-years) | HR (95% CI)* | Events (n) | Incidence rate (per 1000 person-years) | HR (95% CI)* |
| <100 | 14019 | 8.55 | 1(ref.) | | 6693 | 9.12 | 1(ref.) | 7326 | 8.09 | 1(ref.) |
| 100-129 | 28788 | 8.49 | 1.06(1.04,1.08) | | 17989 | 8.27 | 0.96(0.93,0.98) | 10799 | 8.88 | 1.15(1.11,1.18) |
| 130-159 | 36532 | 8.82 | 1.17(1.14,1.19) | | 27002 | 8.52 | 1.04(1.01,1.07) | 9530 | 9.82 | 1.32(1.28,1.36) |
| 160-189 | 27565 | 9.35 | 1.31(1.28,1.34) | | 19935 | 9.00 | 1.17(1.14,1.21) | 7630 | 10.40 | 1.46(1.42,1.51) |
| ≥190 | 20218 | 10.27 | 1.51(1.48,1.55) | | 12477 | 10.19 | 1.42(1.38,1.46) | 7741 | 10.40 | 1.54(1.49,1.59) |
| P for trend |  |  | <0.0001 | |  |  | <0.0001 |  |  | <0.0001 |

HR, hazard ratio; CI, confidence interval

*Adjusted for age, sex, BMI, smoking, alcohol drinking, exercise, income status, use of statins, fasting glucose levels, hypertension, and duration of diabetes

Figure S1. Flow chart of the study population

n = 2,522,388

Subjects (≥ 40years) with diabetes mellitus who underwent **health examinations f**rom January 2009 to December 2012

72,891 subjects were excluded with missing data on at least one variable

80,679 subjects were excluded: having cancer before the index year

291,683 subjects were excluded: having myocardial infarction or stroke before the index year

n = 2,077,135

Subjects eligible for inclusion

**Followed from index year (2009-2012) to the date of incident cardiovascular diseases or until Dec 31, 2017**
